# Influence of ADT on B7-H3 expression during CRPC progression from hormone-naïve prostate cancer

**DOI:** 10.1038/s41417-023-00644-9

**Published:** 2023-07-14

**Authors:** Ning Kang, Hui Xue, Yen-Yi Lin, Xin Dong, Adam Classen, Rebecca Wu, Yuxuan Jin, Dong Lin, Stanislav Volik, Christopher Ong, Martin Gleave, Colin Collins, Yuzhuo Wang

**Affiliations:** 1 Department of Experimental Therapeutics, BC Cancer, Vancouver, BC Canada; 2 Vancouver Prostate Centre, Vancouver, BC Canada; 3 Department of Urologic Sciences, Faculty of Medicine, University of British Columbia, Vancouver, BC Canada; 4 University of Victoria, Victoria, BC Canada

**Keywords:** Prostate cancer, Cancer immunotherapy

## Abstract

Androgen deprivation therapy (ADT) is the standard care for advanced prostate cancer (PCa) patients. Unfortunately, although tumors respond well initially, they enter dormancy and eventually progress to fatal/incurable castration-resistant prostate cancer (CRPC). B7-H3 is a promising new target for PCa immunotherapy. CD276 (B7-H3) gene has a presumptive androgen receptor (AR) binding site, suggesting potential AR regulation. However, the relationship between B7-H3 and AR is controversial. Meanwhile, the expression pattern of B7-H3 following ADT and during CRPC progression is largely unknown, but critically important for identifying patients and determining the optimal timing of B7-H3 targeting immunotherapy. In this study, we performed a longitudinal study using our unique PCa patient-derived xenograft (PDX) models and assessed B7-H3 expression during post-ADT disease progression. We further validated our findings at the clinical level in PCa patient samples. We found that B7-H3 expression was negatively regulated by AR during the early phase of ADT treatment, but positively associated with PCa proliferation during the remainder of disease progression. Our findings suggest its use as a biomarker for diagnosis, prognosis, and ADT treatment response, and the potential of combining ADT and B7-H3 targeting immunotherapy for hormone-naïve PCa treatment to prevent fatal CRPC relapse.

## Introduction

B7-H3, also known as CD276, is a B7 family immune checkpoint molecule first discovered in 2001 [1]. It is universally expressed across various species and is the most evolutionarily conserved B7 family member so far [2]. Despite being initially described as an immune costimulatory molecule [1], later findings showed that B7-H3 is an immune inhibitory molecule affecting T cell activation and proliferation [3].

Importantly, B7-H3 has recently been demonstrated to be overexpressed in various solid tumors but less expressed in normal tissues [4]. Its expression is always associated with advanced disease stages and poor prognosis [5]. Therefore, B7-H3 becomes an attractive target for cancer immunotherapy. Currently, various B7-H3 targeting therapeutics, including B7-H3 vaccines [6], B7-H3-specific antibodies [7], and B7-H3-targeted CAR-T cells [8], are in pre-clinical and clinical studies [9].

B7-H3 has also been reported to be highly expressed in prostate cancer (PCa) [10, 11], suggesting its potential in PCa treatment. Androgen receptor (AR) is the master transcription factor that drives PCa growth and differentiation [12]. Androgen deprivation therapy (ADT) is the standard care for high-risk hormone-naïve PCa patients. But so far, the dynamic changes of B7-H3 expression during PCa progression, particularly its expression following ADT, up to the progression to castration-resistant PCa (CRPC), is largely unknown. It has been reported that AR is a negative regulator of CD276 (B7-H3) gene transcription [13]. Contradictorily, gene expression analysis using various clinical cohorts suggested a positive correlation of B7-H3 with AR and AR activity [13, 14]. Hence, we hypothesize B7-H3 expression is under multiple, complex levels of regulation in PCa, leading to various expression patterns at different stages during CRPC progression.

To select appropriate patients and an ideal time window for B7-H3 targeting therapies, knowing their B7-H3 expression level in PCa tissue is crucial. In this study, we investigated the dynamic changes of B7-H3 expression during CRPC progression from hormone-naïve PCa, following ADT treatment, using a panel of unique CRPC progression models from hormone-naïve PCa patient-derived xenografts (PDXs). These models accurately recapitulate the dynamics of prostate cancer progression and treatment responses found in the clinic [15, 16] and allow for the tracking of B7-H3 expression during PCa progression. Furthermore, we validated our findings in patients’ PCa samples. Our findings may help identify patients and determine the optimal timing of B7-H3 targeting immunotherapies for PCa patients.

## Materials and methods

### Cell lines

Human prostate cancer cell line LNCaP (CRL1740™), C4-2 (CRL-3314™), and PC-3 (CRL-1435™) were obtained from the American Type Culture Collection (ATCC; Manassas, VA, USA). The cell lines were maintained in cell culture dishes in RPMI-1640 (Gibco, ThermoFisher Scientific, Waltham, MA, USA) or Dulbecco’s modified Eagle’s medium (DMEM; Gibco) supplemented with 10% fetal bovine serum (FBS; GE Healthcare HyClone, Chicago, IL, USA) at 37 °C in a humidified incubator with a 5% CO_2_/air atmosphere. Mycoplasma testing was routinely performed using MycoAlert^TM^ mycoplasma detection kit (Lonza, Basel, Switzerland). All the cells were used within a passage range of 5–15. For androgen deprivation treatment (ADT), prostate cancer cell lines were starved with RPMI-1640 containing 5% androgen-depleted charcoal-stripped FBS (CSS; Corning Inc., Corning, NY, USA) or with the addition of 10 uM enzalutamide (ENZ; Sigma-Aldrich, St. Louis, MO). For androgen supplementation treatment, cells were cultured in the 5% CSS medium with the addition of 10 nM 5α-Dihydrotestosterone (DHT; Sigma-Aldrich). Cell viability was determined by trypan blue exclusion.

### PCa patient-derived xenograft (PDX) models

All transplantable PDX tumor lines were grafted under the renal capsule of male NRG mice as previously described [15]. The biological and genetic background of the parental PDX tumor lines is summarized in Supplementary Table S1. Each PDX tumor line was grafted into at least 6 host mice. When tumor reached 300 mm^3^ in volume, 3 mice were sacrificed, and tumors (pre-castration, pre-Cx) were harvested for RNA, protein, and histopathologic analyses. The rest of mice were castrated and monitored for tumor growth. Post-castration (post-Cx) tumors were either collected at 12-week or at different time points post-Cx, with at least 3 mice at each time point. This study followed the ethical guidelines stated in the Declaration of Helsinki, and specimens were obtained from patients with their informed written consent following a protocol (#H09-01628) approved by the Institutional Review Board of the University of British Columbia (UBC). Animal studies were conducted according to protocol #A17-0165, which was approved by UBC’s Animal Care Committee. All PDX tumors were harvested before castration (pre-Cx) or at different time points after host castration (post-Cx) or at CRPC after cancer relapse (CR).

### Human PCa samples and clinical data

PCa specimens, including radical prostatectomy or robotic prostatectomy samples from patients treated with neo-adjuvant hormonal therapy (NHT) and patients treated with surgery alone (control), were obtained from the Vancouver Prostate Centre Tissue Bank. Tissue microarray (TMA) of duplicate 1 mm cores was constructed manually (Beecher Instruments) from post-ADT and control specimens. For cohort 2009, sections from 41 ADT-treated and 21 control group samples remained attached to the TMA slides after B7-H3 or Ki67 immunohistochemical (IHC) staining. For cohort 2011, sections from 38 ADT-treated and 34 control group samples remained attached to the TMA slides after IHC staining and were qualified for analysis. These studies followed the ethical guidelines stated in the Declaration of Helsinki, and specimens were obtained from patients with their informed written consent following a protocol (#H09-01628) approved by the Institutional Review Board of UBC. The baseline characteristics of patient samples are summarized in Supplementary Tables S2 and S3.

The RNA expression data from the High-low clinical cohort were acquired from Dr. Colin Collins [17, 18]. RNA-Seq reads were mapped using STAR 2.6.0a to the reference database built upon human genome sequences and gene annotations from Ensembl GRCh38 Release 90. We obtained the read counts for all genes by htseq-count 0.11.2, and estimated differential expression using DESeq2 1.16.1. Gene expression datasets were uploaded to figshare (https://figshare.com/projects/High-Low_Risk_Prostate_Cancer_Cohort_of_Vancouver_Prostate_Centre/166142). In this study, only data from high-risk patients (Supplementary Table S4) were used for comparing B7-H3 expression between untreated and NHT-treated samples.

### Immunohistochemical (IHC) staining and scoring

Tumor tissue was fixed with formalin and embedded in paraffin. Tissues were sectioned, probed, and stained for IHC analyses as previously described [19, 20]. The following antibodies and conjugates were used: rabbit anti-human B7-H3 (Clone: EPR20115; AbCam, Cambridge, UK; 1:1000), mouse anti-human Ki67 antibody (Clone: MIB-1; Dako, Jena, Germany; 1:25), rabbit anti-human androgen receptor (AR) antibody (Clone: ER179(2); AbCam; 1:100), rabbit anti-human prostate-specific antigen (PSA) antibody (Clone: EP1588Y; AbCam; 1:200), biotinylated goat anti-rabbit antibody (Vector Laboratories, Burlingame, CA, USA; 1:200), and biotinylated goat anti-mouse antibody (Vector Laboratories; 1:200). Rabbit IgG and mouse IgG were used as isotype control antibodies. Human breast cancer tissue was used as B7-H3 staining positive control. For Ki67 staining, images of five random fields at ×400 magnification were taken per tumor and cells were counted to determine the percentage of positively stained cells. For B7-H3 staining, images of five random fields at ×400 magnification were taken per tumor, and staining intensity was assessed by percentage scoring using the following formula: intensity = (% area score 3) × 3 + (% area score 2) × 2 + (% area score 1) × 1.

### Western blotting and quantification

SDS-PAGE and Western blotting were done following the standard protocol. Membranes were probed with rabbit anti-human B7-H3 antibody (Clone: EPR20115; AbCam) or mouse anti-human GAPDH antibody (Clone: GA1R; Invitrogen), and HRP-coupled secondary antibodies prior to the application of ECL reagent (Millipore). The blots were imaged with a Bio-Rad ChemiDoc^TM^ MP imaging system (Bio-Rad, Hercules, CA, USA), and the bands were analyzed with Image Lab software (Bio-Rad).

### Quantitative real-time PCR (qPCR)

Quantitative real-time PCR was performed on an ABI ViiA7 Real-Time PCR System (ThermoFisher Scientific) using TB Green Premix Ex Taq II (TaKaRa, Shiga, Japan). Expression of CD276 transcripts was assessed using previously published primers [21]: CD276-forward: CTCACGAAGCAGGTGAAGCTGCC; CD276-reverse: ACCTACAGCTGCCTGGTGCGC. The primers of endogenous control, human GAPDH (ID: 378404907c1), were selected from PrimerBank database [22]: GAPDH-forward: GGAGCGAGATCCCTCCAAAAT; GAPDH-reverse: GGCTGTTGTCATACTTCTCATGG. PCR products were validated by gel electrophoresis before qPCR analysis. The cycling program consisted of 95 °C for 10 min, followed by 40 cycles of 95 °C for 15 s/60 °C for 1 min. Fold changes were calculated using the 2^−ΔΔCt^ method.

### Statistical analysis

Statistical analyses were performed with GraphPad Prism 7.0 (GraphPad Software). Mean ± SD was used to present the center value and the error bar in the figures. For significance, a minimum *p* value of <0.05 was used as cut-off. For comparison of two means, a two-sided student *t* test was performed. When data was in the form of matched pairs, a paired *t*-test was performed. The association of B7-H3 expression with Ki67 was assessed using Pearson’s correlation.

## Results

### Reduced B7-H3 expression was observed in post-ADT dormant clinical samples and ADT-induced dormant PCa PDX models

**Fig. 1:**
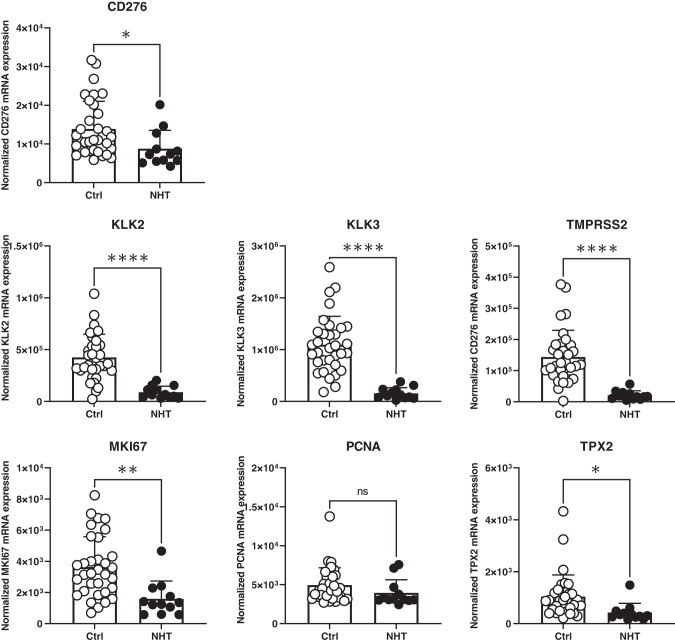
Significant decrease of B7-H3 mRNA expression was observed in post-NHT dormant PCa patient samples. Compared to untreated (Ctrl) group, NHT treated group showed significantly reduced mRNA expression of CD276 (B7-H3), AR activity markers (KLK2, KLK3, and TMPRSS2), and proliferation markers (MKI67, PCNA, and TPX2).

To assess the impact of ADT on B7-H3 expression in hormone-naive prostate adenocarcinoma, we first analyzed CD276 (B7-H3) mRNA expression level using high-risk PCa clinical cohort with 45 samples from radical prostatectomy. High-risk cases were selected for this study by meeting any of the following criteria: Gleason ≥8, PSA ≥ 20, or clinical stage T3a and above. Within the cohort, 12 samples were from neo-adjuvant hormone therapy (NHT) treated patients (patients with prior hormone/androgen deprivation therapy), and the rest were from untreated (Ctrl) patients. As shown in Fig. 1, AR activity was significantly reduced in post-NHT samples as shown by decreased KLK2, KLK3, and TMPRSS2 mRNA expression. Consistently, marked decrease of cell proliferation was also observed, as shown by decreased MKI67, PCNA, and TPX2 mRNA expression. Importantly, CD276 mRNA level was significantly lower in samples from ADT-treated patients when compared with those from untreated patients.

**Fig. 2:**
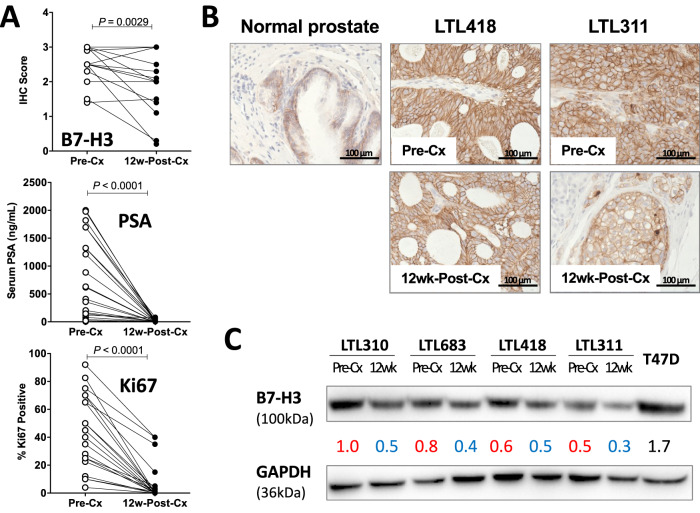
Castration-induced dormant PCa PDXs showed decreased B7-H3 expression compared to active PDXs. **A** B7-H3 IHC scores of 22 pairs of active and castration-induced dormant PDXs. The dormant status was further confirmed by serum PSA level and Ki67 count. A detailed list of PDXs and B7-H3, PSA, and Ki67 changes is shown in Supplementary Table S1. **B** Representative B7-H3 IHC images (×400 magnification) of normal prostate, as well as examples of active (Pre-Cx) and castration-induced dormant (12wk-Post-Cx) PCa PDXs. **C** Representative Western blot analysis of selected PDXs. T47D is a human breast cancer cell line used as a positive control. Castration: Cx.

Next, we performed B7-H3 IHC staining on a group (22 pairs) of pre- and 12-week post-castration (Cx) PCa PDXs to confirm the above findings. At the protein level, 14 out of 22 pairs showed reduced B7-H3 expression in 12-week post-Cx PDXs compared to pre-Cx samples (Fig. 2A, B). The hormonal response and proliferation status of the PDXs were further confirmed with Ki67 staining and serum prostate-specific antigen (PSA) level, and the B7-H3 downregulation in 12-week post-Cx dormant PCa PDXs was confirmed with Western blot analysis (Fig. 2C).

### B7-H3 increased temporarily following castration although decreased during PDX dormancy

**Fig. 3:**
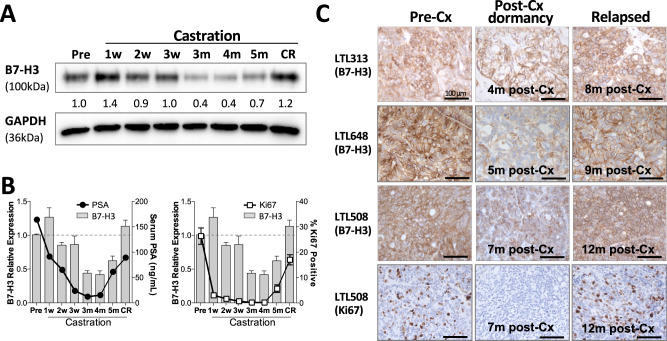
B7-H3 increased temporarily following castration but decreased during PDX dormancy. **A** Time course of Western blot analyses on LTL311 PDXs showed that B7-H3 increased after castration (from 1 to 3 weeks post-castration), gradually decreased and reached the lowest point at 3–5 months post-castration and increased at cancer relapse (CR). **B** LTL311 disease course was confirmed by serum PSA levels and Ki67 counts. **C** Representative B7-H3 IHC images (×400 magnification) from three more PDXs (LTL313, 648, and 508) and Ki67 IHC image from LTL508 during their active and dormant stages as well as at cancer relapse.

We then assessed B7-H3 expression level in the LTL311 PDX model before castration and at various time points after castration (Fig. 3A). Western blot results showed that B7-H3 increased at 1 week post-Cx, dropped to pre-Cx level during 2–3 weeks post-Cx, further decreased during tumor dormancy (3–5 months post-castration), gradually increased at 5 months post-Cx, and eventually rose above pre-Cx level at relapse. LTL311 disease course was confirmed with PSA level and Ki67 counts (Fig. 3B). The reduction of B7-H3 expression during post-ADT PCa dormancy was further confirmed by IHC staining in 3 more PDX models (Fig. 3C).

### B7-H3 expression was negatively regulated by androgen receptor (AR) during the early phase of ADT, but for the rest of PCa disease course, it was AR-irrelevant and proliferation-associated

**Fig. 4:**
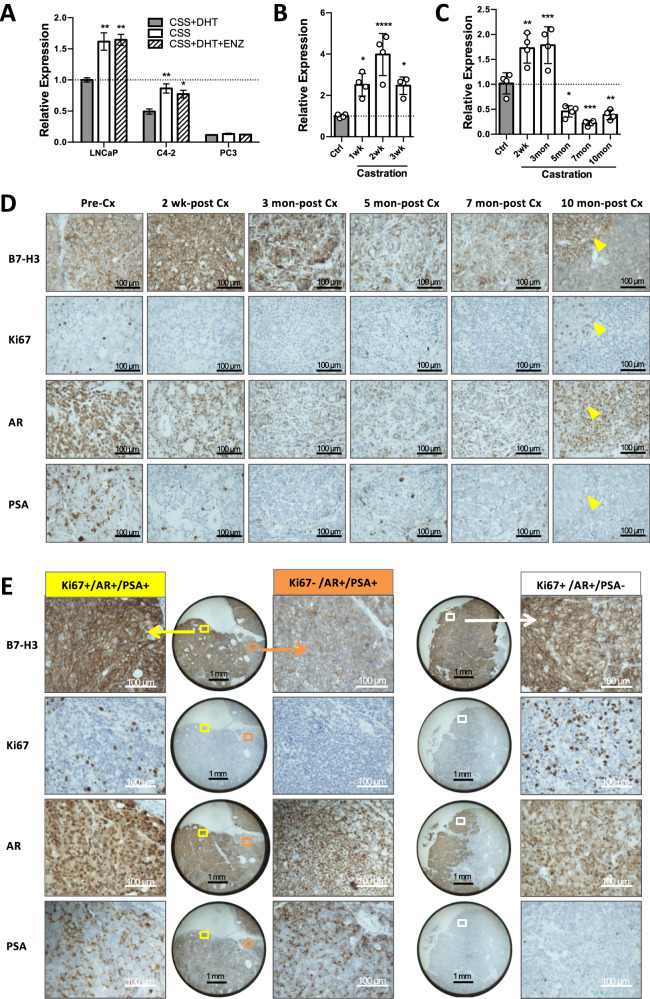
B7-H3 expression was negatively regulated by AR during the early phase of ADT, but positively associated with proliferation beyond that phase. **A**–**C** qPCR B7-H3 transcript analysis demonstrated increased B7-H3 mRNA expression upon androgen deprivation in androgen-sensitive PCa cell lines (LNCaP and C4-2, **A**) and PDXs (LTL674, **B**, and LTL508, **C**). **D** Representative IHC staining images (×400 magnification) of B7-H3, proliferation marker (Ki67), and AR activity markers (AR and PSA) from PDX LTL508 at pre-Cx, 2-week, 3-month, 5-month, 7-month post-Cx, and at early relapse (10-month post-Cx). Yellow arrows indicate sites of tumor recurrence. **E** IHC staining images (×25 and ×400 magnification) of B7-H3, Ki67, AR, and PSA from PDX LTL508 at late relapse (12-month post-Cx).

It has been reported that AR is one of the negative transcriptional regulators of B7-H3 [13]. To further confirm the effect of androgen deprivation on B7-H3 expression, we first used hormone-sensitive PCa cell lines, LNCaP and C4-2, and assessed B7-H3 mRNA expression under androgen-supplemented condition (CSS + DHT) versus androgen deprivation conditions (CSS; CSS + DHT + ENZ). As shown in Fig. 4A, after 1 week of culture under androgen ablation conditions, both androgen-sensitive PCa cell lines (i.e., LNCaP and C4-2) showed significantly increased B7-H3 mRNA expression, compared to those with normal androgen exposures. In contrast, B7-H3 mRNA expression level was not affected by the presence or absence of androgen in the AR-negative and androgen-independent PC3 cell line. This finding was further confirmed in hormone-naïve PCa PDX models. As shown in Fig. 4B, B7-H3 mRNA expression was significantly increased in PDX LTL674 during the first 3 weeks of host castration. For PDX LTL508 (Fig. 4C, D), B7-H3 mRNA and protein expression increased up to 3 months post-castration, decreased during deep dormancy and reached its lowest point at 7-month post-castration. At 10 months post-castration, LTL508 showed signs of early relapse. Clusters of Ki67-positive cells could be seen, and these cells had enlarged nuclei and elevated membranous B7-H3. LTL508 disease course was confirmed by AR and PSA staining. For late relapse at 12-month post-castration (Fig. 4E), three types of tumors could be identified, i.e., Ki67 + /AR + /PSA + , Ki67−/AR + /PSA + , and Ki67 + /AR + /PSA−. B7-H3 upregulation could be seen in Ki67 + /AR + /PSA+ and Ki67 + /AR + /PSA− regions but not in Ki67−/AR + /PSA+ region.

### B7-H3 expression was positively correlated to PCa proliferation status in clinical samples

**Fig. 5:**
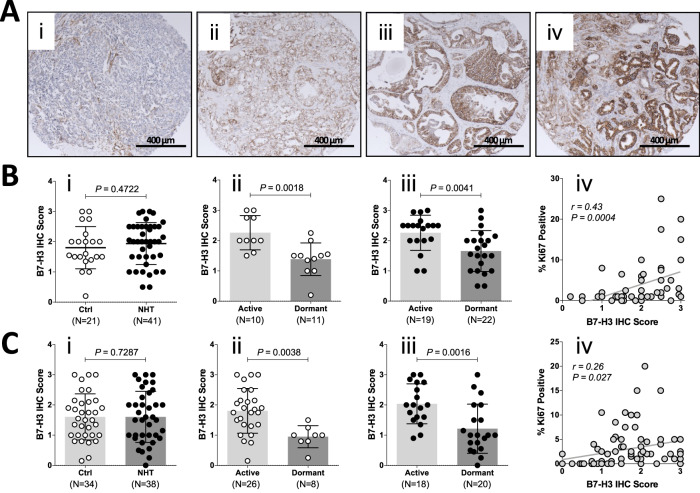
B7-H3 expression was positively correlated to PCa proliferation status and significantly reduced in dormant clinical PCa samples. **A** Representative IHC images showing (i) none, (ii) low, (iii) moderate, and (iv) high B7-H3 expression in clinical PCa samples. **B**,**C** B7-H3 IHC scores of tumor samples from VPC 2009 cohort (**B**) and VPC 2011 cohort (**C**). (i) B7-H3 IHC scores were similar in the untreated (Ctrl) and NHT treated patients. (ii) In the Ctrl group, B7-H3 IHC scores were significantly reduced in dormant patient samples (Ki67 < 1%) compared to active patient samples (Ki67 > 1%). (iii) In the NHT group, B7-H3 IHC scores were also significantly reduced in dormant patient samples compared to active patient samples. (iv) B7-H3 IHC scores were positively correlated to Ki67 percentages in 2009/2011 VPC cohort.

The influence of ADT on B7-H3 protein expression was then studied using radical prostatectomy samples from patients that underwent NHT treatment. Figure 5A showed representative images of B7-H3 IHC staining. For both 2009 VPC cohort (Fig. 5B) and 2011 VPC cohort (Fig. 5C), prostatectomy samples from NHT-treated patients (ADT group) showed similar levels of B7-H3 expression compared to samples from untreated patients (Ctrl group). However, B7-H3 expression was significantly reduced in less-proliferative (dormant; Ki67 < 1%) PCa samples from both untreated and ADT-treated patients. Moreover, B7-H3 protein expression was positively correlated with PCa proliferation status, as shown by Ki67 counts.

## Discussion

PCa is the second most common cancer worldwide and remains one of the leading causes of cancer-related death in men in North America. For high-risk hormone-naïve PCa patients, the mainstay of the therapy continues to be ADT. However, despite an initial response, the vast majority of residual PCa enters a dormant state for months or years and later relapses in the form of incurable CRPC [23, 24]. Progression to CRPC is associated with poor prognosis, impaired quality of life, and fewer therapeutic options. Although there have been significant advances in CRPC therapeutics development in recent years, CRPC, particularly metastatic CRPC, remains a lethal and hard-to-treat disease [16, 23–28]. Therefore, to prevent or delay the development of the fatal CRPC, we propose to develop more effective therapeutic strategies by targeting early critical drivers of lethal CRPC development, while patient’s PCa can still be well managed.

One potential strategy in this regard is immunotherapy. Nowadays, immunotherapies aiming at restoring immunosurveillance and counteracting immune escape mechanisms are revolutionizing the clinical management of a wide range of tumors [29]. Many tumors evade immune surveillance through upregulating immune checkpoint co-inhibitory ligands, like PD-1 ligand 1 (PD-L1) [30, 31], and immune checkpoint inhibitors (ICI) targeting PD-L1/PD-1 axis have become routinely part of the clinical approach for the management of many types of tumors [32].

However, most PCa patients are not responsive to PD-L1/PD-1 blockade therapy [33, 34]. This is due to the unique characteristics of PCa: low PD-L1 expression and naturally “cold” with limited immune cell infiltration [34, 35]. In contrast to PD-L1, immune checkpoint co-inhibitory ligand B7-H3 is preferentially expressed in most PCa compared to benign prostate tissues [11]. In addition, more pieces of evidence suggest that B7-H3 is associated with tumor aggressiveness, migration, and metabolic reprogramming [11, 36, 37]. Thus, beyond immune inhibitory function, B7-H3 may also serve as a specific tumor antigen for advanced PCa management. As a result, B7-H3 seems to be an attractive target for PCa immunotherapy, and various B7-H3 targeting therapeutics have been studied in pre-clinical and clinical trials and have demonstrated their feasibility for clinical application [14, 38–41].

Many factors may affect the outcome of B7-H3 immunotherapies. Adequate B7-H3 expression level is the leading factor. CD276 (B7-H3) gene transcription is negatively regulated by AR [13]. Contradictorily, gene expression analysis using various clinical cohorts suggested a positive correlation of B7-H3 with AR and AR activity [13, 14]. To discover the real relationship between AR and B7-H3 expression and avoid between-individual variations, we performed a longitudinal study on the B7-H3 expression during PCa disease progression using our unique PCa PDX models. Our data suggest a dynamic change and complex regulation of B7-H3 expression during PCa progression and following ADT treatment. It partially agrees with others, that AR is the negative transcriptional regulator of B7-H3 [13], but also suggests AR’s role in B7-H3 regulation is generally recessive and masked by regulators associated with proliferation. During PCa disease course, B7-H3 is predominantly linked to proliferation status: highly proliferative PCa has more B7-H3 expression, whereas dormant PCa has reduced or no B7-H3 expression. This explains why B7-H3 expression positively correlates with AR and AR activity in clinical PCa cohorts [13, 14], where AR activity is positively associated with proliferation and is the driver for hormone-naïve PCa progression. Only at the early phase of androgen deprivation, when PCa cells become less proliferative due to androgen withdrawal, AR-mediated regulation becomes dominant, resulting in less transcriptional inhibition and more B7-H3 expression. Therefore, by using our unique PDX models, we were able to perform longitudinal study and view the dynamic regulation of B7-H3 expression following ADT: B7-H3 expression rises during the early stage of ADT treatment (AR regulation is dominant), gradually drops and reaches its lowest level during ADT-induced tumor dormancy and gradually increases at relapse (proliferation-related regulation is dominant).

Interestingly, in our High-low clinical cohort (Fig. 1), when NHT successfully induced tumor dormancy, B7-H3 expression was significantly reduced compared to untreated group. But in our 2009 VPC and 2011 VPC clinical cohorts, B7-H3 expression level was not affected by NHT. The latter results are consistent with previous findings, which suggest that B7-H3 remains stable in response to hormone therapy [42]. But in fact, these results are not paradoxical. B7-H3 was significantly reduced in the NHT-responsive tumors, post-NHT dormant tumors in this case. Also, the longitudinal study using our PDX models made the repeated observation of B7-H3 expression in the same patient tumor possible and confirmed the findings from the High-low clinical cohort.

The second determining factor for a successful B7-H3 targeting therapy is the tumor immune microenvironment (TIME). Unfortunately, the “cold” PCa TIME remains a potential barrier to successful B7-H3 targeting immunotherapy. To this end, ADT can induce cancer cell death and intratumoral inflammation [43–45], thereby turning “cold” tumors into “hot” tumors. Our data demonstrated that B7-H3 expression could be temporarily but significantly upregulated following ADT treatment, along with the heated-up TIME for attracting more immune cells, the efficacy of B7-H3 targeting immunotherapy might be significantly improved if used in combination with ADT or right after ADT.

In this study, we investigated the dynamic change of B7-H3 during PCa disease course and focused on the influence of ADT on B7-H3 expression. By referring to the changing pattern, it is possible to predict B7-H3 expression level based on the analyses performed on archival biopsy tissue, identify patients, and determine the optimal timing of B7-H3 targeting immunotherapies. Most importantly, our data suggest that B7-H3 might be a promising target for PCa immunotherapy, particularly in the early weeks post-ADT before PCa enters dormancy. Treatment-induced tumor dormancy is a stage in cancer progression where cells cease dividing and survive in an inactive state to avoid being killed by anticancer treatments. Dormant tumor cells are treatment-resistant and capable of re-entering the cell cycle when the unfavorable conditions improve or when they effectively adapt to the new conditions. Although the biological and clinical significance of tumor dormancy is increasingly being recognized, the mechanisms for it are still largely unknown [46, 47]. PCa treated with ADT often enters dormancy. After years of dormancy, PCa can relapse as a more aggressive and castration-resistant fatal disease [18, 23, 24]. Therefore, it is critical to fully kill PCa cells and prevent them from entering dormancy. To this end, B7-H3 targeting therapy, when used in combination with ADT, can more effectively kill PCa cells, lower the chance of dormant cell formation, and reduce the risk of PCa recurrence. Admittedly, our data also indicate B7-H3-targeting therapies are likely irrelevant to ADT-induced dormant PCa, which leaves an interesting question— what are the players responsible for dormant cancer immune evasion?

### Supplementary information

Supplementary table 1

Supplementary table 2

Supplementary table 3

Supplementary table 4

## Data Availability

The authors understand that a submission to the journal implies that materials described in the paper, including all relevant raw data, will be freely available to any researcher willing to use them for non-commercial purposes, without breaching participant confidentiality.
